# Braithwaite’s Retrospect, and Ayer’s Cherry Pectoral, and Cathartic Pills

**Published:** 1857-09

**Authors:** 


*Braithwaite's Retrospect, and Ayer's Chery Pectoral, and Cathartic Pills.*—For three years past one of the pages of the cover of the well-known periodical, *Braithwaite's Retrospect*, republished by Stringer & Townsend, of New York, has been occupied by the advertisements of the two patent medicines manufactured by J. C. Ayers, of Lowell, Mass., and which have been puffed into notice by all the appliances of advertisements, and the full energies of steam presses. These nostrums are now thrust under the very noses of the profession, and almost down their throats.

It required all the impudence of which it is reported that the manufacturer of these two articles possesses, to make the pages of a medical periodical professedly devoted to the advancement of legitimate medicine, the means of extending his sales and increasing the wealth which he is said to have accumulated in their manufacture.

It strikes us, that upon the part of the American publishers of this work, it is rather taking the advantage of the good nature and spirit of forbearance of a profession, which extends to them a generous support in the enterprise in which they are engaged. It is bad enough, to find the daily and weekly secular and *religious* press, engaged in giving notoriety to and puffing every quack remedy of the day. They work simply for money, and justify their course by no other rule of action. The only limits to the boundaries which they set for the exercise of their freedom, is that the language shall be so covert as to not too rudely shock the sense of common decency and propriety. Pills to procure an *abortion* find place with those to cure the ague, *provided,* the advertisement of the former pills contains the transparent piece of gammon of advice, "*that ladies in the third or fourth month of pregnancy are cautioned against taking them as miscarriage will be certain to ensue!*"

But medical men have a right to expect and demand that a higher tone of ethics shall govern the publishers of our professional journals. The profession ignores these remedies in all their forms, and it is not in the province of our *book publishers* to question the motives which actuate medical men in their confirmed hostility to the whole class of quack and patent remedies, and it is but little less than an insult, to use the means which their relation to the profession furnishes them, to thrust upon the notice of the latter the puffs and certificates which constitute the greater part of the stock in trade of the venders of these panaceas.

It furnishes no excuse that the formulæ of the remedies are given, as is the case of the two articles under special notice; this is merely one of the new tricks of the trade introduced comparatively recently. It is only a means proposed for making the profession the agents and venders of these articles. Moreover, we say, with these two receipes before us, that if any regular practitioner of medicine cannot make an *extemporaneous* prescription calculated to meet all the indications of a case, which would be covered by either or both these remedies, he deserves to be *drummed* out of the profession.

We have, perhaps, noticed this subject sufficiently at length, and hope it will be productive of purging the pages of *Braithwaite* from this contamination. If the attention of the profession is once turned to the subject, they will not view the continuance of the practice with complacency, and the only wonder is that it should have been allowed to continue so long without attracting notice, and meeting censure. N.

---

*Editorial Changes.*—Drs. Purple and Buckley have withdrawn from the editorial management of the New York Journal of Medicine, leaving it exclusively in the hands of Dr. Stephen Smith.

